# A Dual-Stream CLIP–ViT Framework for Open-Set Animal Re-Identification: Multi-Seed Ablation, Background-Bias Bracketing, and Query-Time Robustness Analysis

**DOI:** 10.3390/jimaging12080354

**Published:** 2026-08-04

**Authors:** Ivan Melegatti Fernigrini, Bensheng Yun

**Affiliations:** School of Artificial Intelligence, Zhejiang University of Science and Technology (ZUST), Hangzhou 310023, China; yunbsh@zust.edu.cn

**Keywords:** animal re-identification, fine-grained visual recognition, CLIP, vision transformer, deep metric learning

## Abstract

Animal re-identification (Re-ID) asks whether two images show the same individual, a recognition task that fits naturally into applications such as reuniting lost pets with their owners. Existing methods report strong scores, but typically under a single seed, one mask granularity, and no query-time corruption analysis, leaving open whether the gains survive deployment. We propose a hierarchical framework decoupling localisation (a YOLOv8 soft-crop) from identity embedding: a dual-stream network fusing a frozen CLIP ViT-B/16 (learned projection) with a fine-tuned ViT-Base carrying L2-norm part attention, trained under ArcFace. On a combined cat+dog open-set benchmark of 173 identities, it attains Rank-1 0.9742/mAP 0.8597 over three seeds, surpassing a ViT-only ablation by +2.39 Rank-1 and +2.07 mAP. Open-set verification shows all configurations converge near 68% true acceptance at the strictest false-acceptance rate. A background-bias evaluation brackets the embedding’s background reliance between a bounding-box lower bound and a SAM-silhouette upper bound; a manual audit retains the reliable soft crop. A nine-corruption audit identifies down-sampling and motion blur as dominant. On PetFace, the architecture retrieves across 14,716 unseen identities and remains viable in a few-shot regime. A Descriptor Vector Exchange (DVE) extension is Pareto-dominated, traced to the ViT’s coarse feature map and architectural redundancy.

## 1. Introduction

Animal re-identification (Re-ID) asks whether two images depict the *same individual* of a given species rather than *the same species*. It is a fine-grained visual recognition task in which intra-class variability (pose, illumination, expression, and viewpoint) is large and inter-class variability (different individuals of the same breed) is small. Among its applications, the recovery of lost companion animals is one of the most socially consequential: a landmark survey [1] found that roughly one companion dog or cat in seven is lost over a five-year period in the United States, and that about 10% of lost dogs and 25% of lost cats are never reunited with their families. Search remains largely manual—canvassing the neighbourhood, paper flyers, and shelter visits [2]—and microchips help only once an animal is physically scanned at a facility [3], which is not the case for the sightings shared informally on social media [4]. The dominant signal is therefore a photograph, yet no automated system performs individual-level matching from user-submitted “in-the-wild” images.

**Three theoretical gaps.** Deploying Re-ID into this setting requires bridging three gaps that controlled benchmarks tend to hide. (i) *The Clutter Gap*: state-of-the-art Re-ID models are trained and evaluated on pre-cropped subjects but deployment scenarios involve, in many cases, cluttered phone photographs—with complex backgrounds, occluded individuals, poor lighting conditions, etc. Yu et al. [5] show that background overfitting alone can cost 10.2 percentage points of mmAP when the background is removed from both training and testing on ATRW. (ii) *The Pose Alignment Gap*: animals are non-rigid bodies whose appearance changes substantially across postures, and global average pooling conflates body-part contributions. (iii) *The Data Scarcity Gap*: typical lost-pet scenarios provide 1–5 images per individual, below the regime where deep metric learning is comfortable.

**Contributions.** This work proposes a hierarchical two-stage framework that targets the three gaps and then *stress-tests* the resulting embedding under conditions rarely reported jointly in the animal Re-ID literature. Our contributions are:

1. A **dual-stream Stage 2** network that fuses a *frozen* CLIP ViT-B/16 [6] encoder, adapted by a lightweight trainable projection (Stream A), with a fine-tuned ViT-Base/16 [7] carrying L2-norm part attention (Stream B), trained jointly under ArcFace [8] through a single BNNeck [9] embedding, preceded by a YOLOv8 [10] soft-crop Stage 1.
2. A **multi-seed ablation** on a combined cat+dog open-set benchmark (173 test identities) that reports sample mean and standard deviation over three seeds for every ViT-based row, complemented by an *open-set verification protocol* (true acceptance at false-acceptance rates of 0.01 and 0.001) on both the body benchmark and the 14,716-identity PetFace dog test split [11].
3. A **background-bias bracket** that bounds the residual background reliance of the embedding between a coarse bounding-box mask (a reliable lower bound) and a pixel-level silhouette mask obtained by prompting the Segment Anything Model (SAM) ViT-B [12] with a specialised dog/cat YOLOv8 detector; a **manual audit** of the silhouette operator then motivates retaining the reliable soft crop—which never discards the subject—as the Stage 1 front-end for a recall-critical task.
4. A **query-time robustness audit** covering nine controlled corruptions (four positional occluders, motion blur, additive Gaussian noise, brightness reduction, JPEG-quality 20, and 4× down/up-sampling) applied to the query split with the gallery held fixed, providing a deployment-relevant complement to throughput-oriented audits of prior work [13].
5. A **hardened negative result** for a Descriptor Vector Exchange (DVE) [5,14] extension: under full backbone backpropagation it is Pareto-dominated by the base model at seed 42 and at the three-seed mean, and a hyperparameter sweep over descriptor-block depth, loss weight and seed finds no Pareto-superior point. We attribute the outcome to the coarse feature-map resolution of the ViT backbone and to architectural redundancy, not to the data.

## 2. Related Work

**Animal Re-Identification.** Early wildlife Re-ID exploited patterned species: tigers (ATRW [15]), Grevy’s zebras [16], and seals or whale sharks (ALFRE-ID [17]). Schneider et al. [18] showed that Siamese networks can match or exceed human-level identification on several species, and the recent WildlifeReID-10k benchmark [19] consolidates the field around shared evaluation protocols. For domestic animals, PetFace [11] provides over a million AnyFace face crops of 257,484 individuals across 13 families, with ArcFace dominating its loss comparison (51.23% average top-1 accuracy versus 41.88% for Softmax and 9.81% for Center loss). LostPaw [20] targets the lost-pet retrieval task with a single-stream contrastive ViT and reports a held-out $F_{1}$ of 91.08% on 31,860 dogs. OpenAnimals [21] provides a per-axis study of which person Re-ID techniques transfer to animals, finding that square inputs and disabling Random Erasing help, whereas many augmentation and attention add-ons inherited from person Re-ID do not. Yu et al. [5] identify background overfitting as the principal failure mode on ATRW and propose a DVE-based unsupervised part-alignment module on top of a SAM/ISNet foreground-removal pipeline.

**CLIP and prompt learning.** CLIP [6] contrastively aligns an image encoder and a text encoder over 400M web image–text pairs, producing a transferable semantic prior. Naive fine-tuning on small Re-ID datasets erodes that prior; CoOp [22] instead trains a small bank of static context vectors on the text side, and CoCoOp [23] makes those prompts image-conditioned. For animal Re-ID, IndivAID [24] generates image-specific prompts via a Meta-Net and reports consistent gains over CLIP-ReID across eight benchmarks (largest on the pose-variable LionData, +13.95 mAP). Our Stream A is deliberately the most conservative member of this family: a *frozen* CLIP encoder with a single learned projection and *no* prompt learning, with text-side prompt learning and the full IndivAID Meta-Net left as the highest-priority future-work item.

**Deep metric learning.** ArcFace [8] introduces an additive angular margin into the softmax objective and trains class-prototype embeddings on the unit hypersphere; it scales to the ∼85,000 identities of MS1MV2 in the face-recognition literature at m=0.5. Triplet loss [25] requires hard-pair mining; Circle Loss [26] unifies class- and pair-based losses with self-paced weighting; and Multi-Similarity Loss [27] mines informative pairs by jointly weighting positive and negative similarities. The BoT recipe [9] combines ArcFace with a BNNeck, PK sampling, and cosine annealing into a strong person Re-ID baseline that transfers robustly to animals [21].

**Part-based alignment.** Descriptor Vector Exchange (DVE) [14] learns dense pixel-level correspondences across geometrically warped views *without* keypoint supervision. Yu et al. [5] apply it on an SE-ResNet50 backbone and report a Re-ID gain on ATRW from DVE together with multi-identity batch sampling.

## 3. Method

### 3.1. System Overview

**Figure 1:**
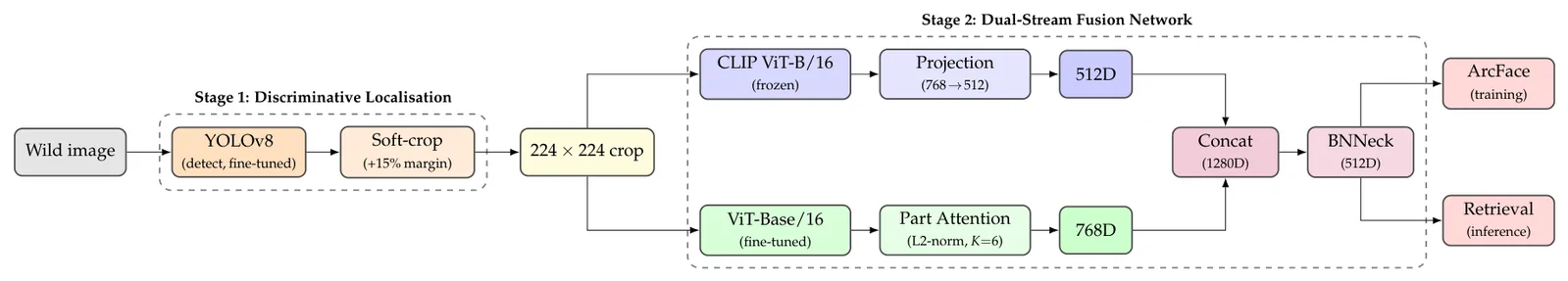
Two-stage pipeline. Stage 1 (YOLOv8 + soft-crop) converts an unconstrained input into a subject-centred 224×224 crop. Stage 2 processes the crop through two parallel streams—a frozen CLIP ViT-B/16 adapted by a learned 768→512 projection, and a fine-tuned ViT-Base with L2-norm part attention—concatenates the 512D and 768D features, and passes them through a BNNeck to a single 512D embedding used by ArcFace (s=64, m=0.5) at training and as the retrieval descriptor at inference.

The system decouples “finding” from “identifying” (Figure 1). Stage 1 applies a YOLOv8 detector [10], initialised from COCO [28] and fine-tuned on the dog/cat classes of OpenImages V7, to localise the subject; the bounding box is expanded by a 15% context margin and cropped (*soft-crop*). If no detection fires, a square centre crop is substituted, so the pipeline always yields a valid input. The detector is deliberately recall-favoured: a missed detection removes the subject entirely and is catastrophic, whereas a loose box is absorbed by the soft-crop margin. YOLOv8 is chosen for this role as a mature, widely supported detector whose accuracy is more than adequate for this localisation stage. Because Stage 1 only has to place a loose, subject-centred box—its errors are absorbed by the 15% margin or the centre-crop fallback rather than propagated as identity errors—detection precision is not the pipeline bottleneck, and a more recent detector (YOLOv9/v10/v11 or an RT-DETR transformer) is a drop-in replacement for Stage 1 that we expect to change little downstream. Because the crop is non-differentiable, it is run offline and cached, which keeps Stage 2 trainable within a modest VRAM budget and lets the same soft-crop be baked into training when the integrated pipeline is evaluated (Section 4.6).

The dual-stream rationale is theoretical as much as empirical: the two streams encode complementary, low-overlap views of identity. Stream A contributes a *class-agnostic* semantic prior learned from web-scale image–text pairs, which addresses the Data Scarcity Gap—a few hundred identities cannot by themselves shape a discriminative feature space, so a transferable prior supplies the structure the data lacks. Stream B contributes a *part-aware* spatial representation whose L2-norm patch attention is sensitive to which body regions are informative, which addresses the Pose Alignment Gap. A single angular-margin objective (ArcFace) then ties both views to the same unit hypersphere, driving every identity towards a maximally separated class prototype. Because the two streams fail on different inputs—the semantic prior on visually near-duplicate breeds, and the spatial stream on uninformative poses—their fusion is complementary rather than redundant; the ablation of Section 4.2 confirms that the fused embedding improves on the stronger single stream.

### 3.2. Stream A: Frozen CLIP Semantic Prior

We use CLIP ViT-B/16 [6] as a frozen image encoder. Its weights are kept frozen throughout training because fine-tuning CLIP on a few hundred identities erodes the very prior that makes it valuable: the gradient signal is too weak to improve the encoder yet strong enough to overwrite it. To adapt the encoder to the identity task without touching the backbone, we attach a single trainable projection—a linear layer followed by layer normalisation—that maps the frozen pooled output to a 512D vector. Only this projection receives gradients. This frozen-backbone, learned-adapter design is strictly less expressive than the prompt-learning family from which it draws motivation (CoOp, CoCoOp, IndivAID); we adopt it deliberately so that the measured contribution of Stream A (Section 4.2) reflects the frozen visual prior alone. It is also the more suitable choice at this data scale: on a benchmark of a few hundred identities the additional capacity of learned prompts risks overfitting—the failure mode CoCoOp [23] was designed to mitigate—whereas a single frozen-backbone adapter is too small to overwrite the transferable prior. Text-side prompt learning and the full IndivAID Meta-Net are therefore left as the highest-priority future extension rather than adopted here.

### 3.3. Stream B: ViT-Base with L2-Norm Part Attention

The second stream is a ViT-Base/16 pre-trained on ImageNet-21K (loaded through timm). Given the 196 output patch features $F\in R^{196\times 768}$ of the final block, we compute the L2 norm $r_{i}={∥f_{i}∥}_{2}$ of each patch as a proxy for its discriminative importance and aggregate the top-K=6 patches by a softmax-weighted average:

(1) $$h_{\mathrm{part}}=\sum_{i\in S}\alpha_{i}\;f_{i},\;\alpha_{i}=\frac{\exp(r_{i})}{\sum_{j\in S}\exp\left(r_{j}\right)},\;\left|S\right|=K.$$

This magnitude-based rule is a deliberately lightweight, parameter-free saliency heuristic; more principled selection criteria, such as PCA- or attention-based part selection, are a natural direction for future work. To retain a global summary alongside this part-level evidence, the part vector is averaged with the classification ([CLS]) token, $h=\frac{1}{2}\left(h_{\left[\mathrm{CLS}\right]}+h_{\mathrm{part}}\right)$. The mechanism adds no parameters and supplies no training signal of its own; all discriminative pressure comes from the ArcFace objective acting on the fused embedding. The ViT backbone is frozen for the first 10 epochs and subsequently fine-tuned at a learning rate 100× lower than the heads ($3\times 10^{-6}$ vs. $3\times 10^{-4}$) to avoid catastrophic forgetting.

A known property of a plain ViT-B/16 is that its patch tokenisation is comparatively weak at capturing fine-grained local textures, edges and small objects, particularly in the early layers—precisely the fine markings on which individual animal recognition depends. The design responds to this on three fronts: the L2-norm part attention above explicitly up-weights the high-magnitude, locally discriminative patch tokens; the DVE extension (Section 4.3) is a direct attempt to add dense local correspondence; and the robustness audit (Section 4.7) confirms and reports transparently that the embedding is indeed most sensitive to losses of high-frequency detail. Pairing an ImageNet-21k-pretrained backbone with the frozen CLIP prior, rather than training the transformer from scratch, is what keeps this weakness from dominating at the present data scale.

### 3.4. Fusion, BNNeck and Loss

The 512D Stream-A and 768D Stream-B features are concatenated into a 1280D vector and passed through a BNNeck [9] that here is a single bottleneck rather than the canonical pre-/post-BN split:

(2) $$e=\mathrm{Dropout}\;\left(\mathrm{ReLU}\;\left(\mathrm{BN}\;\left(Wz\right)\right)\right)\in R^{512}.$$

The L2-normalised embedding e serves a dual role: at training it is the input to ArcFace, and at inference it is the descriptor compared against the gallery by cosine similarity. A single shared embedding suffices because the objective is purely angular—the original split exists only to serve a Euclidean triplet term simultaneously with an angular one. ArcFace is computed against a class-prototype matrix $W\in R^{C\times 512}$:

(3) $$L_{\mathrm{ArcFace}}=-\frac{1}{N}\sum_{i=1}^{N}\log\frac{e^{s\cos(\theta_{y_{i}}+m)}}{e^{s\cos(\theta_{y_{i}}+m)}+\sum_{j\neq y_{i}}e^{s\cos\theta_{j}}},$$

with s=64, m=0.5 on the body benchmark (C=449) and m=0.3, $\mathrm{lr}=1\times 10^{-4}$, an 8-epoch warm-up on the larger PetFace pool (C=3040), which eases early pressure on the more crowded angular space and avoids the degenerate fixed point identified in preliminary runs.

### 3.5. Optional DVE Extension

For the part-alignment ablation, we add a spatial descriptor head (768→128, Linear + LayerNorm) on the ViT block-3 intermediate features and train it jointly with a DVE loss [5,14]. For each patch *u* of an image x and its affinely warped view $x^{\prime }=T\left(x\right)$, descriptor similarities define a soft correspondence whose expected matched position $\hat{g}\left(u\right)$ is regressed to the known warped position T(u):

(4) $$\begin{array}{cc} p(v|u) & ={\mathrm{softmax}}_{v}\;\left(\frac{\langle \Phi {\left(x\right)}_{u},\Phi {\left(x^{\prime }\right)}_{v}\rangle }{\tau }\right),\\ L_{\mathrm{DVE}} & =\frac{1}{N}\sum_{u}{∥\hat{g}\left(u\right)-T\left(u\right)∥}^{2}, \end{array}$$

with $\hat{g}\left(u\right)=\sum_{v}p\left(v|u\right)\;c_{v}$, $\lambda_{\mathrm{DVE}}=0.2$, τ=0.1, and random affine warps ($\pm 30^{\circ }$, scale 0.85–1.15, translation ±10%); the total loss is $L_{\mathrm{ArcFace}}+\lambda_{\mathrm{DVE}}L_{\mathrm{DVE}}$. DVE gradients propagate through the descriptor head *and* through ViT blocks 0–3, as the formulation requires; memory feasibility is restored by gradient checkpointing [29], at the cost of ≈80% additional step time once the backbone is unfrozen. A detached-backbone variant is included as a diagnostic baseline (Section 4.3).

### 3.6. Training Protocol

We train with AdamW (weight decay $5\;\times \;10^{-4}$) under a warm-up cosine schedule whose rate rises linearly from 20% to 100% of its target over epochs 0–4, PK sampling (P=10, K=4, batch 40), BF16 mixed precision, and early stopping (patience 15 evaluations) on a *combined* cat+dog validation Rank-1. RandomErasing is deliberately disabled, following the OpenAnimals finding [21] that it removes exactly the subtle local markings (stripes, spots, facial patterns) that discriminate individuals; a square input geometry is kept for the same animal-oriented reasons. We depart from the OpenAnimals ARBase recipe on a single axis: a pretrained ViT-B/16 backbone rather than an IBN-ResNet-50.

## 4. Experiments

### 4.1. Datasets and Protocols

**Figure 2:**
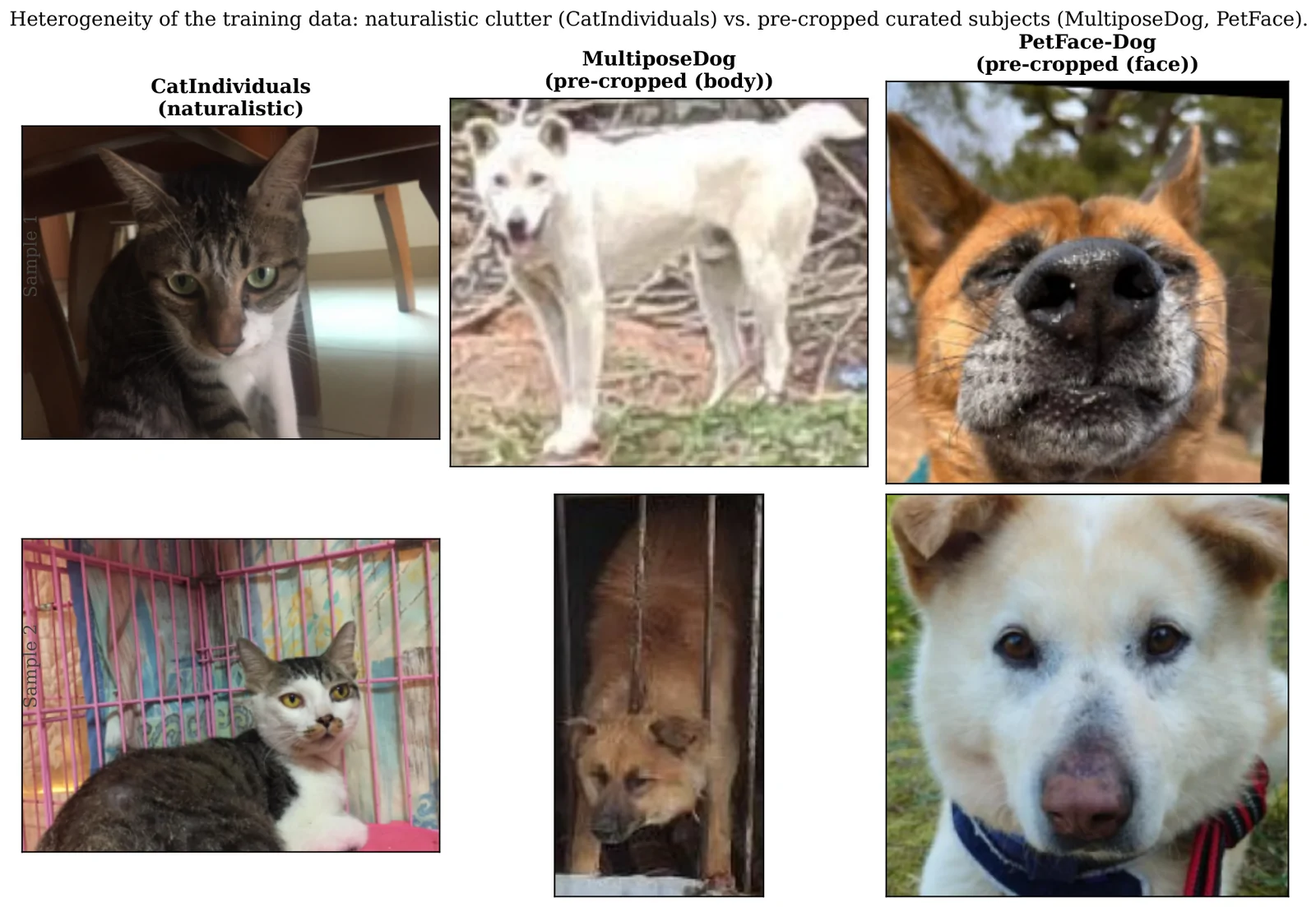
Representative samples from the three training datasets. **Left** (CatIndividuals): naturalistic phone photographs with substantial clutter—furniture, cages, secondary animals, occluding objects, human limbs. **Middle** (MultiposeDog): tight Re-ID-style body crops in multiple poses. **Right** (PetFace): face-only crops from the AnyFace detector. CatIndividuals dominates body Re-ID training (79% of identities, 91% of images) and provides the in-the-wild signal; MultiposeDog and PetFace contribute pre-cropped curated samples. The CatIndividuals [30] and MultiposeDog [31] samples are reproduced under the CC BY 4.0 licence; the PetFace [11] samples are reproduced with permission from the dataset creators.

We use three datasets spanning a deliberate naturalistic-to-curated spectrum (Table 1, Figure 2). CatIndividuals [30] (354 train/75 val/77 test identities; 9192 training images) is the principal source of *in-the-wild* training signal: user-submitted phone photographs containing furniture, secondary animals, hands and occluding objects, and the dataset closest in distribution to the queries a lost-pet service would actually receive. MultiposeDog [31] (95 train/96 test; Market-1501 format) supplies tight, pre-cropped body crops with substantial pose variation, and the dog subset of PetFace [11] supplies AnyFace face crops. The combined cat+dog *body* benchmark pools the MultiposeDog query/gallery (96 dog identities) with the held-out CatIndividuals test split (77 cat identities), for 173 identities and 181 query/2319 gallery images; validation pools the MultiposeDog query/gallery with the 75-identity CatIndividuals validation split, so checkpoint selection reflects both species. All evaluations are open-set with respect to identity (train and test identity sets disjoint) and closed-gallery at the retrieval level (each query has at least one ground-truth match).

We report Rank-*k* accuracy (Cumulative Matching Characteristic, CMC) and mean Average Precision (mAP). Of the two, mAP is the primary metric for overall retrieval quality, while Rank-5 is the application-facing metric for the lost-pet task, where an owner inspecting the top-5 results has a high probability of finding the pet.

### 4.2. Multi-Seed Ablation on Body Re-ID

We ablate four architectural conditions on the combined benchmark and report each ViT-based row as the *multi-seed* mean and sample standard deviation over three independent runs with seeds {7,42,123}, alongside the seed-42 single-run value for context (Table 2). All other hyperparameters are identical across rows.

Three findings emerge.

**ResNet→ViT is the largest step.** Replacing the convolutional backbone with a ViT-Base lifts the seed-42 Rank-1 by +8.29 and mAP by +15.26 percentage points (+9.7% and +22.7% in relative terms); the ResNet baseline also peaks early in validation and then degrades under continued angular-margin pressure, whereas the transformer remains stable. It is true that convolutional networks can outperform ViTs on small datasets, but this applies to *from-scratch* training [7]; here the ViT is ImageNet-21k-pretrained and paired with the frozen CLIP prior—the transfer regime in which transformers are strong—so the convolutional baseline (row A) does not overturn it.

**The CLIP→ViT gain survives variance.** The difference of multi-seed means between C and B is +2.39 Rank-1 and +2.07 mAP percentage points (≈+2.5% relative on each metric), both above the pooled standard deviation, and the Rank-1 advantage holds in *each* of the three seeds individually (C > B at seeds 7, 42 and 123: 0.9669/0.9834/0.9724 against 0.9503/0.9337/0.9669); the contribution is not an artefact of a favourable seed. With only three seeds a formal significance test is underpowered, so we report the sample dispersion (a 95% confidence interval follows as $\mu \pm 4.30\;\sigma /\sqrt{3}$) and this per-seed consistency rather than a *p*-value the sample cannot support. The frozen CLIP prior is also associated with a lower Rank-1 seed variance across these three runs (sample standard deviation 0.0166→0.0083); as with the mean difference we read this as indicative rather than established, since three seeds do not support a formal variance comparison—indeed it does not carry over to the verification Area Under the Curve (AUC, Section 4.4). The apparent stabilisation is in any case specific to the top-1 decision: the mAP variance is not reduced (0.0165 for ViT-only against 0.0185 for CLIP + ViT). We report the two effects separately rather than folding them into a single “CLIP is better” claim. For a deployed matcher the frozen CLIP prior nonetheless earns its place: a small but seed-consistent top-1 gain at negligible added cost, with the owner-facing top-5 reaching a mean of 0.9871.

**The DVE extension is Pareto-dominated.** Row D sits below the base CLIP + ViT on both metrics at seed 42 (0.9669/0.8523 vs. 0.9834/0.8731) and at the three-seed mean (0.9669±0.0055/0.8513±0.0029 vs. 0.9742±0.0083/0.8597±0.0185); its only favourable property is a tighter seed distribution. We analyse this negative result next.

### 4.3. DVE: A Hardened Negative Result

A natural concern is whether the DVE outcome is an artefact of one hyperparameter choice. With seed 42 fixed and all other settings as in Table 2, we sweep the ViT block whose patch tokens feed the descriptor head {3,6,9} and the loss weight $\lambda_{\mathrm{DVE}}\in \left\{0.05,0.1,0.2,0.5,1.0\right\}$. (With a ViT backbone the token grid stays at 14×14 across the depth sweep, so the axis varied is the descriptor’s receptive-field depth, not its spatial resolution as in Yu et al.’s SE-ResNet adaptation.) Across all three depths and all five loss weights—combined with the three seeds at the canonical (block3, λ=0.2) point—no configuration is Pareto-superior to the base: λ=0.2 and λ=0.5 tie for the sweep’s highest Rank-1 (0.9669), and although λ=0.5 attains the sweep’s best mAP (0.8651) it still trails the base (0.8731).

We attribute the result to two architectural factors rather than to the data. First, *feature-map resolution*: DVE learns dense per-position correspondences and was validated where the SE-ResNet map is downsampled only 4× (56×56), with a modest reported gain of +2.0 to +2.4 mmAP on ATRW; our ViT-B/16 exposes a 14×14 token grid—about sixteen times fewer spatial positions—far coarser than the regime in which DVE was shown to help. Second, *architectural redundancy*: the frozen CLIP prior (Stream A) and the L2-norm part attention (Stream B) already aggregate the discriminative regions a correspondence objective is meant to align. Dataset scale is *not* the differentiator—our 449-identity pool exceeds the ATRW Re-ID split (∼107 training entities). A faithful test of DVE on a higher-resolution feature map (a finer token grid or a convolutional backbone) is identified as future work. The detached-backbone variant performs no better and isolates a methodological lesson: detaching the backbone silently disables the very mechanism DVE is designed to apply. For deployment this settles the question: on the coarse ViT token grid, the dense-correspondence objective adds training cost without improving retrieval, so the base dual-stream is the model we retain.

### 4.4. Open-Set Verification

Retrieval metrics presuppose that the query has a match in the gallery. The deployment scenario is structurally different: most queries received by a lost-pet service have no match, and the operational quantity of interest is the cost-weighted error rate of a yes/no decision at a chosen similarity threshold. We construct class-balanced positive and negative pairs from each query/gallery split (RNG =42; impostors restricted to the same species), score them by cosine similarity of the L2-normalised 512D embeddings, and report the AUC, Equal Error Rate (EER), and True Acceptance Rate (TAR) at two False Acceptance Rate (FAR) operating points, FAR =0.01 and 0.001 (Table 3).

Three observations follow. **CLIP leads at the moderate operating points:** CLIP + ViT (C) attains the best AUC (0.9860), the lowest EER (5.52%) and the best TAR at FAR =0.01 (84.63%, +4.11 pp over ViT-only). **The configurations converge at the strictest FAR:** at FAR =0.001—the regime most representative of a deployed matcher—all three body models sit within about a point of one another (67.6–68.7% TAR), with DVE marginally highest, and the gaps are smaller than the per-seed standard deviations, so no model holds a robust low-FAR advantage. **The CLIP variance reduction does not carry over:** the AUC seed standard deviation is in fact largest for CLIP + ViT (0.0053 vs. 0.0024 for ViT-only), so the stabilising effect seen on retrieval Rank-1 is specific to that decision and does not extend to the threshold-based scores. The single-seed face checkpoint reaches 74.38% TAR at FAR =0.001 over an 85× larger gallery, indicating that the face-cropped pipeline scales better into the low-FAR regime than the body pipeline. For a live matcher, which must run at a strict FAR, the chosen operating threshold rather than the backbone therefore governs the missed-match rate, and a face-cropped gallery is the better substrate in that regime.

### 4.5. Open-Set Generalisation at Scale and the Few-Shot Regime

“At scale” here qualifies evaluation, not training. We train the same CLIP + ViT architecture on the PetFace dog filtered set (3040 IDs/16,335 imgs; m=0.3, an 8-epoch warm-up) and evaluate on the dog test split: 14,716 unseen identities, each contributing one query and one gallery image. The model reaches Rank-1=0.5414, Rank-5=0.6933, mAp=0.6132—a test gallery nearly two orders of magnitude larger than the body benchmark (173 vs. 14,716 identities) in a strict open-set regime. We deliberately make no state-of-the-art claim against the full 46,755-identity PetFace training benchmark, since we train on a filtered subset. These open-set retrieval numbers are not directly comparable to the *seen-identity* top-1 accuracies tabulated by Shinoda and Shiohara [11] (whose ArcFace baseline averages 51.23% across families, with 77.86% for dogs under independent per-family training), which evaluate individuals also present in training. A protocol-matched comparison is nonetheless available on the *open-set verification* track, which PetFace scores as AUC over unseen identities: our PetFace face model reaches 98.93% AUC on the same 14,716-identity dog verification split (Section 4.4), about half a point below their full-training-set ArcFace baseline (99.45%) and above their dog dataset-transfer baselines (ImageNet 97.18%, MegaDescriptor 93.75%, CLIP 91.86%), despite our ∼15× smaller filtered training subset.

The filtered baseline excludes precisely the identities that motivate the Data Scarcity Gap. We therefore re-train under a relaxed ≥2 images-per-identity filter with K=2 images per identity per batch (P=20 so the batch size stays 40), expanding the pool to 11,471 IDs/41,073 imgs (median 3 images per identity, 90.2% in the 2–5 range). The result is Rank-1=0.5885, Rank-5=0.7386, mAp=0.6589, exceeding the filtered baseline by +4.71 Rank-1 and +4.57 mAP percentage points *despite* a 3.77× larger and substantially sparser training pool. The defensive filter is therefore unnecessary on this benchmark: a deployed service can enrol a pet from as few as two reference images and still retrieve across nearly 15,000 unseen identities. The truly singular case (K=1, a single reference image) is harder in kind rather than only in degree: it removes the within-batch positive pair on which angular-margin metric learning depends—with one image per identity there is no second same-identity sample to attract—and is left as future work.

### 4.6. Background-Bias and the Stage-1 Choice

Stage 1 retains a 15% context margin rather than a tight cut-out. We ask two coupled questions: how much does the Stage 2 embedding rely on the background the soft crop leaves in frame, and is the suppression operator one we can trust as a front-end on real photographs? Each test image (query and gallery) is re-rendered under three masking conditions—*original*; *bounding-box* (everything outside the YOLO box filled with the ImageNet-mean colour; reproducible, but it leaves the rectangular ring of background inside the box); and *SAM silhouette* (the box prompts SAM to remove the in-box background to the animal outline). We cross this with three training regimes (base, soft-crop, silhouette), giving the 3×3 matrix of Table 4.

The base model fixes the magnitude. Removing only the out-of-box background costs it just −1.65 Rank-1 and −4.80 mAP, whereas removing the in-box background as well costs −19.9 and −28.8. The true reliance of the deployed soft crop is therefore bracketed between the two: the 15% margin removes very little of the cue the model uses, but an apparent in-box dependence survives that the soft crop does not reach—an upper bound, as the audit below shows. The direction matches Yu et al. [5], whose comparable train-original/test-masked transition costs −19.2 mmAP; our upper-bound drop is of the same order. Reading down the columns, suppressing the background at training time reduces the reliance monotonically (the original→SAM gap shrinks from −19.9 to −18.2 to −14.9 Rank-1), but at a cost in clean accuracy (the silhouette-trained model loses −1.65 Rank-1 on unmasked images, against −0.55 for soft-crop training). The mechanism is not a simple background story. Because the SAM mask is imperfect—the audit below finds it clips identity-relevant content in about a fifth of images—silhouette training fits the embedding partly to degraded crops, which transfers slightly worse to clean images; by the same token the −19.9 Rank-1 upper bound conflates lost background with lost subject rather than isolating background reliance alone.

**Figure 3:**
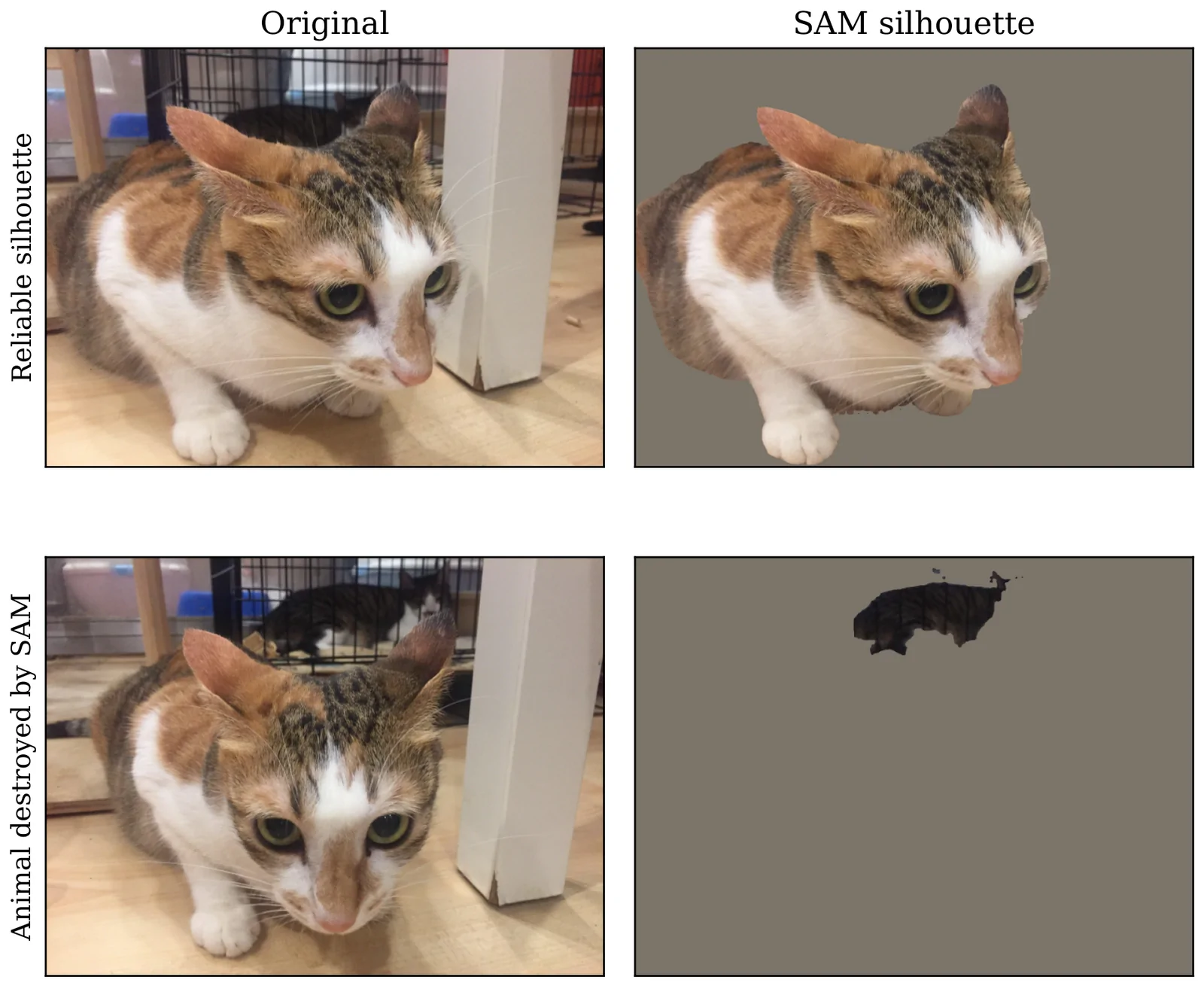
SAM foreground segmentation on close-up cat photographs is unreliable. **Top**: a clean silhouette. **Bottom**: a failure case—prompted by the YOLO box, SAM locks onto a background object and erases the foreground cat. A manual audit of 400 images finds the silhouette removes identity-relevant content in about one in five and erases the animal outright in ∼4%, which is why the reliable soft crop, not silhouette segmentation, is adopted as Stage 1. The cat photographs are from CatIndividuals [30], reproduced under the CC BY 4.0 licence.

On the matrix alone the silhouette regime would win. That verdict does not survive an audit of the masking operator. What matters for re-identification is the loss of *discriminative* content (eyes, nose, ears, coat markings) rather than pixel area. No automated proxy we tried matches human judgement on this: a whole-frame grey fraction, an interior-hole fraction and an embedding-shift score all reach AUC ≤0.60 for predicting human-judged damage. We therefore measure the effect by manual audit. On a uniform random sample of 400 training images, the silhouette removes identity-relevant content in 20.8% of the images (95% CI 17.1–25.0) and erases the subject outright in 4.2% (2.7–6.7); see Figure 3. The failure is intrinsic to SAM on close-up pet imagery—the polarity is correct, the cat is removed and the background kept—and cannot be screened at inference without a second segmenter. Closing this failure mode is a segmentation problem in its own right: multi-point or box-ensemble SAM prompting to disambiguate the subject from a salient background object, or a pet-specialised segmenter fine-tuned against this close-up failure case, are the concrete routes to a foreground stage reliable enough to deploy. For a recall-critical task a destroyed query cannot be recovered downstream, so the front-end must be judged against a conservative reliability bar. The soft crop clears it. Built with the YOLO soft crop, the training images fall back to a safe centre crop for only 0.73% of images and *never* remove the subject. Baking it into the pipeline reaches 0.9669 Rank-1 (train and test both soft-cropped), only −1.65 below the no-Stage-1 figure of 0.9834 (the base/original cell of Table 4)—the same small drop as the bounding-box bound. Foreground segmentation is therefore *not* adopted as a deployment stage. The silhouette regime is retained only as a cautionary upper-bound ablation—the more so because its mask artefacts are image-deterministic, so a silhouette-trained, silhouette-tested model can latch onto them.

**Fallback on the evaluation set.** Quantified on the combined test set, the recall-favoured soft crop falls back to a centre crop for 6/181 query images (3.31%; MultiposeDog 4.81%, CatIndividuals 1.30%) and 13/2319 gallery images (0.56%). Conditioning retrieval on the Stage-1 outcome, the 175 non-fallback queries reach Rank-1=0.971 (mAp=0.876) while the six fallback queries reach Rank-1=0.667 (mAp=0.578); with only six such queries we report the figures without over-interpreting them. Because a fallback returns the whole image rather than a crop—and so never amputates the subject—a fallback query degrades to the no-Stage-1 condition rather than failing outright, consistent with the recall-favoured design. The practical upshot for a deployed matcher is that the background reliance it must live with is bounded and mostly out-of-box: the reliable soft crop removes that part at a small cost, while the larger in-box reliance is reachable only through a segmenter too unreliable to deploy.

### 4.7. Query-Time Robustness Audit

The deployment scenario is asymmetric: shelter gallery images are typically curated, but owner queries are not. We embed the unperturbed combined gallery once and re-embed the 181 combined query images (104 dog + 77 cat) under nine controlled corruptions, recomputing the metrics against the same gallery (Table 5; NumPy seed 42 for the stochastic perturbations). Single-run figures should be read against the multi-seed noise floor of $\sigma_{R_{1}}=0.0083$ for this model.

The two most damaging corruptions are both capture-time losses of high-frequency detail: a 4× down/up-sampling pass (−16.0 Rank-1) and a 15-pixel horizontal motion blur (−13.3 Rank-1, and the worst single mAP drop at −14.0). Both remove the fine markings a ViT embedding leans on without changing global appearance, and both cost more than any occluder—though both are milder than the SAM background-removal upper bound of Section 4.6, which deletes background *and* part of the subject. Occlusion is strongly position-dependent but not head-biased on this combined benchmark: the left and centre quarters (which cover the subject’s core for the portrait-framed cats) cost most (−6.63 Rank-1, and −9.18 mAP for the centre), while the bottom quarter costs least. The embedding is, by contrast, robust to photometric and storage artefacts (brightness halving −2.76, JPEG-q20 −4.42). The cheapest mitigation is a client-side pre-flight check that rejects low-resolution queries and warns on detected motion blur—more cheaply than a wider augmentation regime, which would conflict with the rationale for disabling RandomErasing. This is complementary to throughput-oriented edge audits such as RAPID [13]: rather than optimising frame rate, we map which corruptions a deep embedding is most sensitive to. The actionable finding is that down-sampling and motion blur dominate the query-side risk, so a client-side minimum-resolution and motion-blur pre-flight check recovers most of the lost Rank-1 at near-zero cost.

### 4.8. Loss-Function Comparison

To validate the choice of ArcFace, we re-train the proposed architecture under three alternative metric losses with all other components fixed, each at the hyperparameters recommended in its originating paper (Table 6).

No paper-default alternative Pareto-dominates ArcFace. Batch-hard Triplet is the closest, only −1.10 Rank-1 and −0.53 mAP behind and in fact winning Rank-5 (0.9945); it is competitive but not Pareto-superior. Circle Loss is next (−2.21/−2.34); its trajectory is healthy, so the gap measures absolute headroom at the animal-Re-ID configuration we adopt from Yu et al. (γ=64, m=0.25) rather than at any of the three task-specific regimes in the Circle Loss paper (γ=256, m=0.25 for face; γ=128, m=0.25 for person Re-ID; γ=80, m=0.4 for fine-grained retrieval). Multi-Similarity, at its CUB/Cars defaults (α=2, β=50), is misaligned with our pretrained-ViT, PK setup—its steep negative-pair gradient pulls apart already-discriminative features—and is the clear outlier. Closing these gaps would require per-benchmark loss-hyperparameter retuning; we retain ArcFace as the canonical loss, with batch-hard Triplet as the only near-equal alternative, so no loss change is warranted for the deployed system.

### 4.9. Comparison with the Literature

We situate the framework in prose rather than a single accuracy table, because the nearest systems are each evaluated on a different benchmark—and, in one case, a different task—so a shared Rank-1/mAP column would invite a comparison the protocols do not support. OpenAnimals [21] reports on its four wildlife benchmarks, IndivAID [24] on ATRW and seven further animal datasets, and LostPaw [20] on its own AdoptAPet dog corpus. LostPaw, the only published system targeting the same lost-pet retrieval task, is moreover not measured in the same currency: it reports a pairwise-verification $F_{1}$ at a fixed threshold rather than a closed-set Rank-1/mAP, so even its headline cannot be placed directly against ours; the cross-evaluation that would make the two commensurable is set out as future work. What can be stated internally is that the 173-identity body benchmark is a relatively small open-set evaluation on which the proposed model’s Rank-5 is high but short of saturation, and that the 14,716-identity PetFace evaluation supplies the larger, harder gallery that exposes the remaining headroom. We deliberately do not benchmark on ATRW [15]: its tiger visual statistics differ substantially from the dog/cat domain, and a cross-species generalisation experiment was scoped out at the planning stage. Readers seeking an ATRW benchmark for a comparable CLIP-based method can find one in IndivAID [24].

### 4.10. Implementation and Reproducibility

All models are implemented in PyTorch 2.x with timm 1.0.24 and HuggingFace transformers, trained in BF16 mixed precision on a single NVIDIA RTX 5070 Laptop GPU (Blackwell, CUDA 12.8, 8 GB). Combined body Re-ID training (449 IDs, 100 epochs with early stopping) takes ≈28 min; PetFace training (3040 IDs, 48 epochs) takes ≈1 h 15 min; the DVE variant adds ≈80% step time after the backbone unfreezes. At inference, the YOLO soft crop is applied only to unconstrained query photographs; gallery embeddings are computed once and cached, so each query costs one forward pass plus a dot-product search, and an optional fast $O(Nk_{1})$ variant of *k*-reciprocal re-ranking [32] ($k_{1}=20$) refines the ranking without the full $O(N^{2})$ encoding.

**Computational cost.** Table 7 quantifies the complete pipeline on the deployment GPU. We report *inference-active* parameters: of the 237.3 M parameters stored in the Stage-2 checkpoint, the frozen CLIP text tower (64.1 M) and the training-only ArcFace classifier never execute at inference, leaving 173.1 M active in Stage 2 and 199.0 M across the full pipeline once the YOLOv8 detector (25.9 M) is included. A single-query forward pass costs 15.4 ms in Stage 2 and 11.2 ms in Stage 1, for 26.6 ms (37.7 queries/s) end to end with the gallery cached; offline gallery encoding runs at 97.4 images/s in batches of 64. Peak GPU memory stays between 1.1 and 1.7 GB, well within the 8 GB budget, and Stage 2 costs 168.6 GFLOPs per image at batch 1. The 196-token ViT-B/16 grid keeps self-attention modest, so it is not the wall-clock bottleneck; the Stage-1 detection and Stage-2 forward passes are what dominate.

## 5. Discussion

**Where the framework succeeds.** The CLIP + ViT model’s +2.39 Rank-1 and +2.07 mAP gains over a ViT-only ablation (≈+2.5% relative on each)—the Rank-1 advantage replicated in all three seeds—support the design rationale of Stream A as a class-agnostic regulariser rather than a magnifier of raw discriminative power. The few-shot retraining probe shows the architecture remains viable in the genuinely sparse two-images-per-identity regime that motivates the Data Scarcity Gap, and the large-scale PetFace evaluation shows the embedding space transfers to 14,716 unseen identities.

**Where it fails, and why.** The hardened DVE negative result is a property of the method-and-architecture combination, not of the method alone: the dense-correspondence objective needs a high-resolution feature map and non-redundant structure to act on, and the 14×14 ViT token grid combined with the CLIP and part-attention priors provides neither. We deliberately do *not* attribute the result to a lack of pose variation in the data—CatIndividuals is cluttered and in-the-wild and MultiposeDog carries substantial pose variation—nor to dataset scale, which exceeds the ATRW split on which DVE was shown to help.

**Recall governs the deployment choices.** Two of our measurements are decided by the asymmetric cost structure of lost-pet retrieval, where a missed reunion is far costlier than a spurious candidate. The background-bias study shows that aggressive foreground segmentation buys the best background robustness but, by a manual audit, defaces or erases the subject in a non-trivial fraction of cats; for a recall-critical front-end we therefore keep the reliable soft crop, which never discards the subject, and treat silhouette suppression as a cautionary ablation. The robustness audit similarly turns into a concrete, cheap pre-flight check (minimum resolution, motion-blur warning) rather than a heavier augmentation regime.

**Methodological defaults.** Both the multi-seed protocol and the open-set verification were inexpensive on a single modest GPU, and both changed the reading of the results: a single-row best would have over-stated the DVE extension and the AUC summary alone would have hidden the low-FAR regime where deployment actually operates. We suggest reporting both as defaults in animal Re-ID.

**Generalisation to further species and settings.** The benchmark already spans two species—cats and dogs—through a single species-agnostic embedding and a Stage-1 detector that routes both, so the framework is not specialised to one kind of animal. Extending it to further companion species (for example rabbits or horses) or to more diverse capture settings is plausible but is not claimed here. As the ATRW tiger case makes explicit, visual statistics differ substantially across animal groups—coat patterning, body plan and the discriminative cue itself all change—so cross-species transfer must be measured rather than assumed; a multi-species benchmark, routed to species-specific galleries by the Stage-1 detector, is the natural way to test it and is scoped as future work.

**Limitations.** (i) Stream A uses no prompt learning; integrating the IndivAID Meta-Net (which reports gains up to +13.95 mAP over CLIP-ReID, and whose own ablation shows the symmetric text-to-image loss *hurts* Stage-2, 16.25→16.07 mAP on NyalaData) is the highest-priority architectural extension. (ii) The 173-identity body benchmark is approaching saturation; an in-the-wild dataset of hundreds to thousands of open-set identities would provide a more discriminative ranking signal. (iii) The single-image-per-identity (K=1) regime is not evaluated; as noted in Section 4.5 it removes the within-batch positive pair that angular-margin training relies on and would need a different enrolment strategy, such as a small support set or a verification-only threshold. (iv) A robust foreground segmenter—one guarded against the close-up failure mode documented above, rather than segmentation per se—and an active-learning loop such as Ambiguity-Aware Sampling [33] (which matches supervised accuracy while labelling 0.033% of pairs) are the natural paths to a low-annotation deployable system. (v) A direct cross-evaluation with LostPaw [20], whose code and checkpoint are public, is the most informative external validation. (vi) Our hyperparameters are set by literature defaults and manual sweeps (the DVE depth/λ and loss-function studies above); automated hyperparameter optimisation is a natural methodological extension, from evolutionary architecture and hyperparameter search [34] to recent bio-inspired metaheuristics [35]. (vii) The ViT-B/16 backbone is comparatively weak at fine-grained local textures and edges; the frozen CLIP prior and the L2-norm part attention mitigate but do not remove this, and it remains visible in the down-sampling and motion-blur sensitivity reported in Section 4.7. (viii) Stage 1 relies on a single YOLOv8 detector held deliberately in a recall-favoured role; a more recent or pet-specialised alternative has not been separately benchmarked.

**Societal context.** The framework targets a problem of real social scale: roughly one companion dog or cat in seven is lost over a five-year period in the United States [1], the dominant search signal is a photograph, and the missing piece is automated individual-level matching from owner-submitted images. Treating this as the actual deployment scenario—rather than an abstract benchmark—motivates every measurement reported here: the verification protocol because most queries have no gallery match, the background-bias bracket because the deployment-time distribution shift removes the most learnable confound, and the robustness audit because phone photographs are asymmetrically degraded relative to curated gallery images.

## 6. Conclusions

We presented a hierarchical dual-stream framework for open-set animal Re-ID that fuses a frozen CLIP ViT-B/16, adapted by a learned projection, with a fine-tuned ViT-Base carrying L2-norm part attention, preceded by a YOLOv8 soft-crop localisation stage. On a combined cat+dog open-set benchmark of 173 identities it reaches Rank-1=0.9834/mAp=0.8731 at seed 42 and 0.9742±0.0083 over three seeds, with the CLIP contribution surviving multi-seed analysis at +2.39 Rank-1 and +2.07 mAP; it scales to 14,716 unseen PetFace identities and remains viable in a genuine few-shot regime. A background-bias bracket and a manual audit of the silhouette operator motivate keeping the reliable soft crop as the recall-critical front-end; a nine-corruption audit identifies down-sampling and motion blur as the dominant query-side vulnerabilities with cheap mitigations; and a DVE extension is a hardened negative result, Pareto-dominated across seeds and a hyperparameter sweep and traced to feature-map resolution and architectural redundancy. The framework takes a concrete step towards a deployable lost-pet retrieval system at the scale at which the problem occurs in practice.

## Figures and Tables

**Table 1:** Dataset statistics. The combined body benchmark merges CatIndividuals + MultiposeDog into 449 training identities; the PetFace dog test split is open-set against either the filtered (3040-ID) or the few-shot (11,471-ID) training subsets.

Dataset	IDs Train	Imgs Train	IDs Test	Use
CatIndividuals	354	9192	77	Body train + val/test
MultiposeDog	95	921	96	Body train + val/test
**Body merged**	**449**	**10,113**	**173**	
PetFace-Dog (≥5)	3040	16,335	14,716	Face Re-ID
PetFace-Dog (≥2)	11,471	41,073	14,716	Few-shot real

**Table 2:** Multi-seed ablation on the combined cat+dog body Re-ID benchmark (173 test identities, open-set). ViT-based rows (B, C, D) report mean ± sample standard deviation over three seeds {7,42,123}; italicised rows are the seed-42 single-run values for context (the ResNet-50 baseline A is seed-42 only). Bold: best multi-seed column.

Model	Rank-1 (μ±σ)	Rank-5 (μ±σ)	mAP (μ±σ)
A: ResNet-50 + BNNeck + ArcFace	*0.8508* (seed 42)	*0.9448*	*0.6714*
B: ViT-Base + BNNeck + ArcFace	0.9503±0.0166	0.9816±0.0128	0.8390±0.0165
* (seed 42)*	*0.9337*	*0.9669*	*0.8240*
C: CLIP + ViT (proposed)	0.9742±0.0083	0.9871±0.0032	0.8597±0.0185
* (seed 42)*	*0.9834*	*0.9834*	*0.8731*
D: CLIP + ViT + DVE (full backprop)	0.9669±0.0055	0.9853±0.0115	0.8513±0.0029
* (seed 42)*	*0.9669*	*0.9724*	*0.8523*

**Table 3:** Open-set verification metrics. Multi-seed rows (B, C, D) report mean ± sample standard deviation over three seeds; the face baseline is a single-seed PetFace dog checkpoint. Italicised rows label the two evaluation tracks. Bold: best column among the body-Re-ID multi-seed rows.

Configuration	AUC	EER	TAR@FAR = 0.01	TAR@FAR = 0.001
*Body Re-ID (combined cat+dog; 96 dog + 77 cat = 173 identities)*				
B: ViT-only (3 seeds)	0.9809±0.0024	6.83±0.41%	80.52±2.80%	67.86±2.11%
C: CLIP + ViT (3 seeds)	0.9860±0.0053	5.52±1.30%	84.63±3.50%	67.63±3.44%
D: CLIP + ViT + DVE (3 seeds)	0.9835±0.0012	5.78±0.36%	84.16±0.86%	68.71±1.33%
*Face Re-ID (PetFace dog test, 14,716 identities, single seed)*				
face baseline	0.9893	4.15%	90.30%	74.38%

**Table 4:** Background-bias matrix on the combined cat+dog benchmark (173 identities). Each cell is Rank-1/mAp. Rows are training-time background regimes; the three test-time columns re-mask the combined query and gallery. The base-model row (top) brackets the embedding’s background reliance: removing only the out-of-box background (*Bbox*, a reliable *lower* bound) versus removing the in-box background as well (*SAM silhouette*, an *upper* bound that also absorbs the segmentation failures audited in the text). Δ_orig→SAM_ is the original→silhouette drop in percentage points; the bracket endpoints are set in **bold**.

Training Regime	Test-Time Mask (Rank-1/mAP)			Δ_orig→SAM_ (*R*_1_/mAP)
	Original	Bbox (Lower)	SAM Sil. (Upper)	
base (raw)	0.9834/0.8731	**0.9669/0.8251**	**0.7845/0.5851**	**−19.9/−28.8**
soft-crop (Stage 1)	0.9779/0.8737	0.9337/0.8255	0.7956/0.5968	−18.2/−27.7
silhouette (cautionary)	0.9669/0.8344	0.9448/0.8007	0.8177/0.6547	−14.9/−18.0

**Table 5:** Query-time robustness on the combined cat+dog benchmark (gallery fixed). Deltas in percentage points relative to the unperturbed baseline. Bold: two worst single-source corruptions.

Perturbation	Rank-1	Δ*R*_1_ (pp)	ΔmAP (pp)
none (baseline)	0.9834	—	—
top25 occluder	0.9337	−4.97	−4.20
bot25 occluder	0.9558	−2.76	−2.66
left25 occluder	0.9171	−6.63	−4.50
cen25 occluder	0.9337	−4.97	−9.18
**motion blur (15px)**	0.8508	−13.26	−13.98
Gaussian noise (σ=0.10)	0.8950	−8.84	−8.10
low light (×0.5)	0.9558	−2.76	−2.65
**low resolution (4×)**	0.8232	−16.02	−12.76
JPEG-q20	0.9392	−4.42	−4.07

**Table 6:** Loss-function ablation on the combined cat+dog body Re-ID benchmark (seed 42). Δ columns are signed differences with respect to ArcFace. Bold: best per column.

Loss	Config	$R_{1}$	$R_{5}$	Δ*R*_1_	ΔmAP
ArcFace (ref.)	s=64, m=0.5	**0.9834**	0.9834	—	—
Circle Loss	γ=64, m=0.25	0.9613	0.9779	−2.21	−2.34
Multi-Similarity	α=2, β=50	0.9061	0.9558	−7.73	−15.94
Triplet (b-hard)	m=0.3, cosine	0.9724	**0.9945**	−1.10	−0.53

**Table 7:** Computational cost of the complete pipeline on the RTX 5070 Laptop GPU. Parameters are inference-active: the frozen CLIP text tower (64.1 M) and the training-only ArcFace head are excluded (the on-disk Stage-2 total is 237.3 M). Stage-2 latency is batch 1 (single-query deployment); batched throughput uses batch 64 (offline gallery encoding).

Quantity	Value
Stage-1 parameters (YOLOv8)	25.9 M
Stage-2 parameters, active	173.1 M
Pipeline parameters, active	199.0 M
Stage-2 FLOPs (batch 1)	168.6 G
Stage-1 latency (detect + soft crop)	11.2 ms
Stage-2 latency (batch 1)	15.4 ms
End-to-end per query (gallery cached)	26.6 ms (37.7/s)
Batched throughput (batch 64)	97.4 img/s
Peak GPU memory (batch 1/64)	1104/1685 MB

## Data Availability

The data analysed in this study were derived from the following third-party resources: the CatIndividuals dataset [30], available in the public domain at https://www.kaggle.com/datasets/timost1234/cat-individuals (accessed on 27 July 2026) under the CC BY 4.0 licence; the Multi-pose Dog dataset [31], available in the public domain at https://doi.org/10.17632/v5j6m8dzhv.1 (accessed on 27 July 2026) under the CC BY 4.0 licence; and the PetFace dataset [11], available at https://dahlian00.github.io/PetFacePage/ (accessed on 27 July 2026) for non-commercial research use upon request to its creators. The trained models and source code are available from the corresponding author upon reasonable request.
